# A Systematic Review on Traditional Uses, Sources, Phytochemistry, Pharmacology, Pharmacokinetics, and Toxicity of Fritillariae Cirrhosae Bulbus

**DOI:** 10.1155/2020/1536534

**Published:** 2020-11-12

**Authors:** Ting Chen, Furong Zhong, Cheng Yao, Jia Chen, Yiqing Xiang, Jijing Dong, Zhuyun Yan, Yuntong Ma

**Affiliations:** College of Pharmacy, Chengdu University of Traditional Chinese Medicine, Chengdu 611137, Sichuan, China

## Abstract

Fritillariae Cirrhosae Bulbus (known as chuanbeimu in Chinese, FCB) is a famous folk medicine which has been widely used to relieve cough and eliminate phlegm for thousands of years in China. The medicine originates from dried bulbs of six species of *Fritillaria* which are distributed in the temperate zone of the Northern Hemisphere. Increasing attention has been paid to FCB because of its excellent medicinal value such as being antitussive, expectorant, analgesic, anticancer, anti-inflammatory, and antioxidative. During the past years, a large number of research studies have been conducted to investigate the phytochemistry, pharmacology, and pharmacokinetics of FCB. A range of compounds have been isolated and identified from FCB, including alkaloids, saponins, nucleosides, organic acids, terpenoids, and sterols. Among them, alkaloids as the main active ingredient have been illustrated to exert significant therapeutic effects on many diseases such as cancer, acute lung injury, chronic obstructive pulmonary disease, asthma, Parkinson's disease, and diabetes. Due to the excellent medical value and low toxicity, FCB has a huge market all over the world and triggers a growing enthusiasm among researchers. However, there is still a lack of systematic review. Hence, in this work, we reviewed the FCB-based articles published in Sci Finder, Web of Science, PubMed, Google Scholar, CNKI, and other databases in the recent years. The traditional uses, sources, phytochemistry, pharmacology, pharmacokinetics, and toxicity of FCB were discussed in the review, which aims to provide a reference for further development and utilization of FCB.

## 1. Introduction

Fritillariae Cirrhosae Bulbus (FCB) is one of the best known Chinese herbal medicines used to treat respiratory disease and obtained from the *Fritillaria* species [1–3]. According to the Chinese pharmacopoeia (2015), there are six species of the *Fritillaria* genus used as the botanical origins of FCB, including *Fritillaria cirrhosa* D. Don, *Fritillaria przewalskii* Maxim., *Fritillaria unibracteata* Hsiao et K. C. Hsia, *Fritillaria delavayi* Franch., *Fritillaria taipaiensis* P. Y. Li, and *Fritillaria unibracteata* Hsiao et K. C. Hsia var. *wabuensis* (S. Y. Tang et S. C. Yue) Z. D. Liu., S. Wang et S. C. Chen (hereinafter to be referred as FC, FP, FU, FD, FT, and FW) [4]. The six *Fritillaria* plants are characterized by poor growth and strict environmental requirements. Also, they are mainly distributed between the altitudes of 3000 and 5000 m in most parts of Tibet, Northwest Sichuan, Northern Yunnan, and Southeastern Qinghai [5, 6].

Historically, FCB was firstly documented in “Shen Nong Ben Cao Jing” (a classic medical book of the Han dynasty) and has been widely used clinically owing to its remarkable antitussive, expectorant, and antiasthmatic activities [7]. So far, over 200 products containing FCB in the market have been applied for the treatment and prevention of a variety of diseases such as cough and asthma [8]. As the development of separation and identification technique, numerous phytochemical investigations concerning FCB have been carried out during the past several decades. Also, many constituents derived from FCB have been identified, including steroidal alkaloids, saponins, terpenoids, and glycosides [9]. Among them, steroidal alkaloids have been regarded as the indicator agent for evaluating the quality of FCB [10]. Recently, increasing pharmacological evidence indicates that FCB has a wide range of pharmaceutical properties, such as expectorant, antitussive, antiasthma, anti-inflammatory, antitumor, and antioxidant properties [11–13], which lay the groundwork for further clinical applications of FCB and trigger a growing enthusiasm among researchers for the ancient herb. Nevertheless, there is no systematic summary of the modern study findings of FCB. Herein, we provided a systematic review on the traditional uses, sources, phytochemistry, pharmacology, pharmacokinetics, and toxicity of FCB in this work, to provide a reference for further development and utilization of FCB.

## 2. Traditional Uses

FCB is one of the most ancient Traditional Chinese Medicine (TCM) associated with high economic and medical value [14, 15]. In China, it has been used as a classical antitussive and expectorant agent for thousands of years [16]. According to historical materials, before the Ming dynasty, FCB and other Fritillaria Bulbs such as Fritillariae Thunbergii Bulbus (known as zhebeimu, FTB) were collectively named “Beimu.” Beimu was firstly recorded in “Shen Nong Ben Cao Jing,” the earliest monograph of TCM, and has been described as a cough medicine with good throat clearing and detoxification effects [7]. Until Ming dynasty, another medical monograph called “Ben Cao Hui Yan” (Nizhumo, 1624, Ming dynasty) has summarized the differences between FCB and FTB, which indicated that FCB tended to moisten the lung, eliminate phlegm, and relieve cough and asthma and FTB tended to clear heat and detoxify. As the research moves along, medical workers have gained a deeper insight into the usages of FCB in the Qing dynasty. “Yao Xing Qie Yong” (Xudachun, 1741, Qing dynasty) has complementally noted that FCB has antidepressant effects. Moreover, “Ben Cao Gang Mu Shi Yi” (Zhaoxuemin, 1765, Qing dynasty) definitely indicated the differences between FCB and FTB on the treatment of cough for the first time. The author thought that FCB and FTB were suitable for the cough results from deficiency and heat, respectively, which is consistent with the modern application of FCB and FTB [17–19]. Of course, some modern medical books also summarized the pharmacology of FCB. For instance, “Zhong Yao Da Ci Dian” described that FCB had a wide spectrum of pharmacological properties, such as clearing heat, moistening lung, eliminating phlegm, relieving cough, and good therapeutic effects on respiratory and pulmonary disease [20]. Generally, FCB is routinely used to grind powder and dissolve it in water before taking it orally. Additionally, FCB as an important ingredient appears in many Chinese patent medicines (CPM) which have been made into various dosage forms, such as pills, ointments, powders, tablets, solutions, and syrups. There are 38 CPM containing FCB documented in Chinese Pharmacopoeia (2015) alone (Table 1). A well-known example is “Chuanbei Pipa Tangjiang.” It has been wildly used to relieve cough and eliminate phlegm [4, 21, 22]. As the increasing studies of pharmacology of FCB, we have a deeper understanding of the role of FCB in human health. Emerging evidence has shown that FCB possesses diverse biological activities, such as expectorant, antitussive, antiasthma, anti-inflammatory, antitumor, and antioxidant activities [10, 23].

## 3. Sources

**Figure 1:**
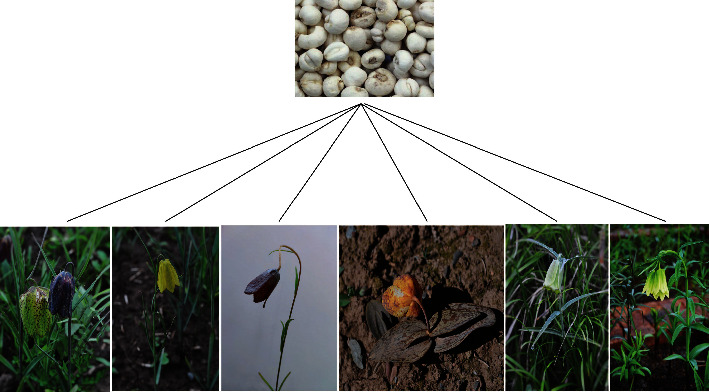
The pictures of FCB (A), FC (B), FP (C), FU (D), FD (E), FT (F), and FW (G).

**Figure 2:**
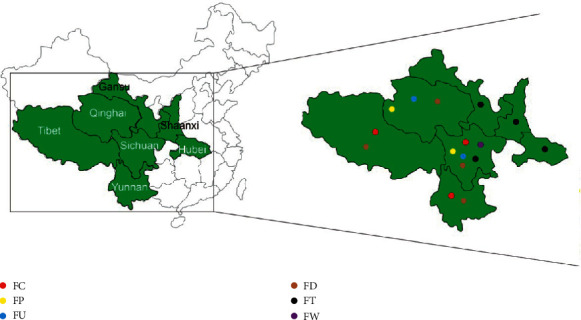
The distribution of the original plants of FCB in China.

According to the Chinese Pharmacopoeia (2015), FCB is derived from the dried bulbs of six *Fritillaria* species, including FC, FP, FU, FD, FT, and FW [4] (Figure 1). It is worth noting that these *Fritillaria* species are generally perennial herbs and have strict requirements for the growing environment. Among them, FC commonly grows in high altitude habitats from 3200–4200 m, has a disjunct distribution across the high mountain areas of Southern and Eastern Tibet, northwestern Yunnan, and western Sichuan. FP is mainly distributed at altitudes between 2800 and 4400 m in Southern Gansu, eastern and southern Qinghai, and western Sichuan. FU usually grows at the altitudes between 3200 and 4500 m in northwest of Sichuan and southeastern of Qinghai. FD is chiefly distributed at the altitudes between 3800 and 4700 m in northwestern Yunnan, western Sichuan, southern Qinghai, and Tibet. FT primarily grows at the altitudes between 2400 and 3150 m in Shaanxi (Qinling Mountains and its south), southeastern Gansu, northeastern Sichuan, and northwestern Hubei [24]. FW is mainly distributed at altitudes between 2500 and 3600 m in northwestern Sichuan [25] (Figure 2). Based on the difference in botanical origins and producing areas, FCB is divided into different types (including songbei, qingbei, and lubei) which have different efficacies and prices. It has long been acknowledged that songbei has the highest medicinal value and excellent quality [26, 27]. In the recent years, the enhancement of health consciousness has induced an increase of the demand of FCB. Also, over 2000 tonnes of FCB was used in China each year [8]. The production of medicinal preparations containing FCB was an industry with an estimated value of US $400 million per year [28]. However, the sources of wild FCB is inadequate for the increasing domestic and international market demand because the original plants of wild FCB grow extremely slow, and they usually require, at least, five years to grow into an apparent size [21]. As a result, extensive commercial use and limited wild stocks lead to a high price of FCB. From 2013 to 2016, the price of FCB has reached 3000 Yuan (RMB) per kg [14]. Terribly, the huge market demand and high profit of FCB further exacerbated the overexploitation of wild sources. Nowadays, these used species in *Fritillaria* genus have been classified as precious, rare, and threatened species by the Endangered Species Scientific Commission of China in 2012 [28].

## 4. Phytochemistry

The earliest research concerning the chemical composition of *Fritillaria* species could date back to 1888. In this year, imperialine, an alkaloid, was firstly isolated from *F*. *imperialis* [29]. Subsequently, extensive chemical studies have been conducted on various *Fritillaria* species, especially on the TCM herbal chuaneimu. To date, more than 100 chemical components (mainly including alkaloids, saponins, nucleosides, organic acids, terpenoids, and sterols) have been extracted and identified from FCB (shown in Table 2).

### 4.1. Alkaloids

**Figure 3:**
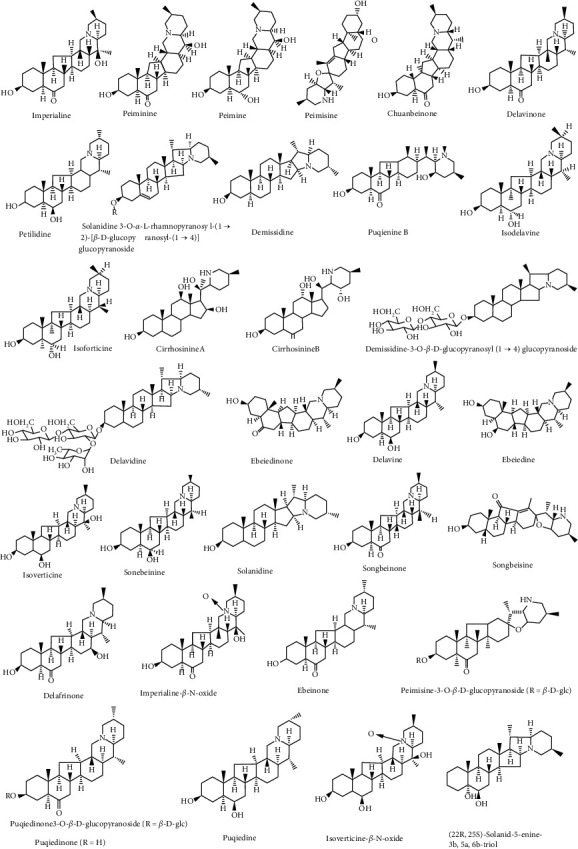
The chemical structure of alkaloids from FCB.

Alkaloids are a group of naturally occurring chemical compounds that contain cyclic structures with, at least, one basic nitrogen atom being incorporated within, which have a wide distribution in medicinal plants [39]. As the fundamental components of FCB, alkaloids are traditionally believed to be responsible for the pharmacological activities of FCB [34] and have been considered as a key index for the identification and quality control of FCB [36]. Interestingly, the content of total alkaloids in FCB is extremely low, approximately ranging from 0.02% to 0.3% [40]. Previous studies showed that the alkaloids from FCB could be classified into two groups based on the carbon framework: isosteroidal alkaloids and steroidal alkaloids; the former accounts for approximately 75% of the total alkaloids [18, 41]. In the last several decades, many phytochemical research studies have been carried out to extract and identify the alkaloids in FCB. To date, more than 30 alkaloids in FCB have been identified, mainly including imperialine, peimisine, peiminine, peimine, and chuanbeinone.  Figure 3 shows the details of that.

### 4.2. Saponins

The saponins are natural surface-active glycosides existing in many popular medicinal plants, which have been regarded as a kind of promising chemical component because of its various pharmacological properties, such as immunomodulatory, antioxidative, antidiabetic, and anti-cancer properties [42]. In the recent years, some studies reported that saponins may be one of the active ingredients responsible for the pharmacological activities of FCB. The content of total saponins in FCB is approximately ranging from 3% to 4% [43]. However, a few research studies focused on the recognition of saponins.

### 4.3. Nucleosides

**Figure 4:**
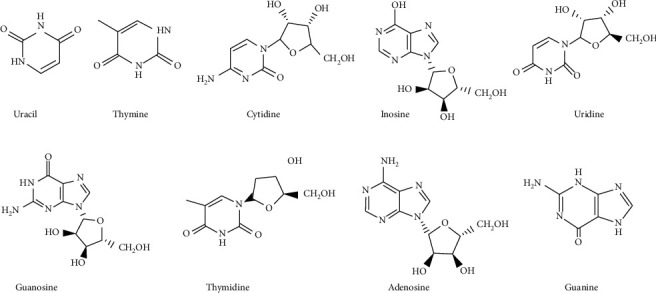
The chemical structure of nucleosides from FCB.

Nucleosides belong to a class of organic compounds with a nitrogen-containing heterocyclic nucleobase and a 5-carbon sugar [44]. They have attracted attention because of their multiple pharmacological effects, such as antitumor, antithrombotic, anti-inflammatory, and anti-fungal effects [45]. Recent chemical investigations suggested that nucleosides are present in the water extract of Fritillaria Bulbs [36], which indicates that nucleosides may participant in the pharmacological activities of FCB. To date, more than 10 nucleosides have been isolated and identified from FCB, such as uracil, thymine, cytidine, inosine, and thymidine. The details of that are shown in Figure 4.

### 4.4. Organic Acids

**Figure 5:**
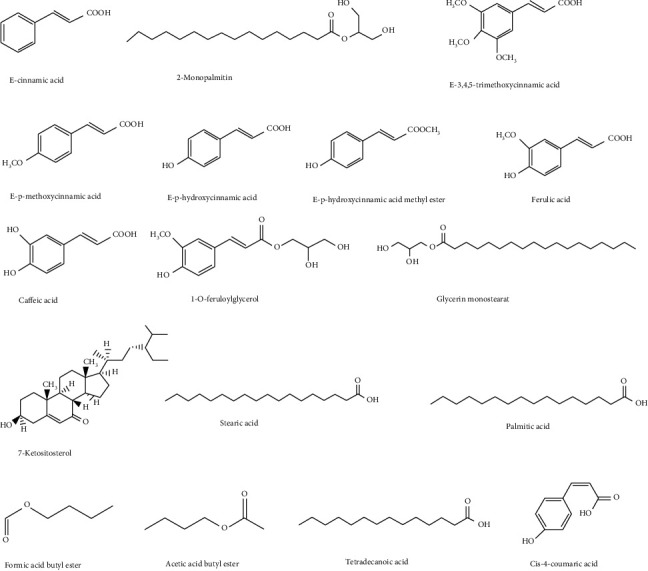
The chemical structure of organic acids from FCB.

Organic acids are also a kind of important compounds existing in FCB. At present, a variety of organic acids have been derived from FCB, and the chemical structure is shown in Figure 5.

### 4.5. Sterols and Terpenoids

**Figure 6:**
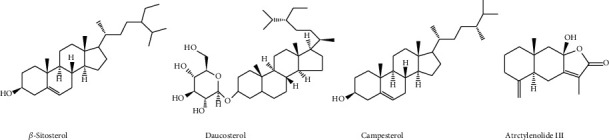
The chemical structure of sterols and terpenoids from FCB.

In addition to alkaloids, saponins, nucleosides, and organic acids, sterols and terpenoids also have been found in many *Fritillaria* species (Figure 6).

### 4.6. Other Compounds

Some compounds from the volatile oil of FCB have been isolated and identified, such as 1-octadecene, 1-dodecene, oxirane, hexadecyl, 1-hexadecanol, 1-eicosanol, 9-octadecynoic acid, methyl ester, and n-hexadecanoic acid [46]. In addition, some elements (Ca, Mg, K, Fe, Co, Ni, Mn, Ba, Ti, A1, Sn, Cr, and Sr) have also been found to exist in FCB [18].

## 5. Pharmacology

**Figure 7:**
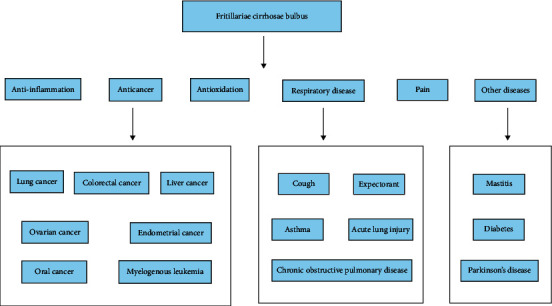
The pharmacological effects of FCB.

Recently, FCB is attracting more and more attention because of its excellent medicinal value and extremely low toxicity. Based on the evidence of previous in vivo or in vitro studies, FCB exerts a series of biological activities, including antitussive, expectorant, analgesic, anticancer, anti-inflammatory, and antioxidative activities, which is summarized in Figure 7 and Table 3.

### 5.1. Anticancer

Cancer is a formidable disease with high morbidity and mortality [75]. Currently, there are several remedies, mainly including chemotherapy, surgery, and radiation therapy. However, severe adverse effects caused by these remedies are unavoidable [76]. Therefore, it is an extreme active domain to search for the anticancer agents with higher bioactivity and lower toxicity in the future. Interestingly, concern pharmacologic studies have indicated that FCB is a potential candidate in the treatment of several types of cancers, such as lung cancer, colorectal cancer, liver cancer, endometrial cancer, oral cancer, ovarian cancer, and myelogenous leukemia.

#### 5.1.1. Lung Cancer

Currently, lung cancer as the most common cancer is attracting increasing attention [77]. In previous studies, FCB or its active compounds have shown significant inhibitory effects on the development of lung cancer. One in vivo study suggested that the total alkaloids of FCB (TFCB) (10–40 mg/kg/day for10 days) markedly suppressed the growth of transplantable Lewis lung carcinoma (LLC) tumor in mice through inhibiting tumor angiogenesis and inducing apoptosis. The molecular mechanism was related to the downregulation of endothelial cell adhesion molecule-1 (CD31) and caspase-3. Furthermore, the antitumor effects of TFCB were also confirmed by an in vitro study. It was observed that TFCB (30 *μ*g/mL for 0–72 h) exhibited inhibitory effects on the proliferation activities of LLC cells, which may partly result from the apoptosis induction mediated by S-phase cell cycle arrest [47]. Besides, accumulated evidence pointed out that the aqueous extract of FCB (FC-AE) also has antitumor effects similar to those of TFCB. It was demonstrated that FC-AE (0.2 mL/2.5 mg/mL/2 day for 20 days) performed suppression activities on the growth of transplantable A549 tumors in a nude mice model, which was associated with the enhancement of signal transducer and activator of transcription (STAT) 1, STAT4, interferon gamma (IFN*γ*), interleukin- (IL-) 12, caspase-3, B-cell lymphoma-2-associated X apoptosis regulator (Bax) levels, and the decrease of B-cell lymphoma-2 (Bcl-2) production. Moreover, FC-AE (0–100 *μ*g/mL for 48 h) exhibited excellent antiproliferative effects on A549 cells, which was attributable to the accumulation of G2 phase cells and the promotion of Bax, STAT1, and STAT4 activities, as well as the downregulation of Bcl-2 proteins [48]. In addition to total extracts, some monomer chemical compositions, such as chuanbeinone and imperialine, also possess huge therapeutic potential on the treatment of lung carcinoma. For example, chuanbeinone (5–15 *μ*g/mL for 48 h) dramatically reduced the viability of LLC cells, and the positive results were accompanied with an accumulation of cells in the S phase and a decrease of the cells in the G0/G1 phase, which subsequently leads to the augmentation of apoptosis. The underlying mechanism was related to the reduction of antiapoptotic protein Bcl-2 and the enhancement of proapoptotic protein Bax and caspase-3. To better understand the role of chuanbeinone on the treatment of lung cancer, an in vivo study suggested that chuanbeinone (10–40 mg/kg/day for 10 days) significantly suppressed the growth of transplantable LLC tumors in mice. The antagonistic effects were attributed to inactivation of CD31-mediated tumor angiogenesis, as well as the promotion of caspase-3-mediated apoptosis [49]. Similarly, extensive studies have demonstrated the antitumor effects of imperialine. A recent study showed that imperialine (200 ng/mLfor 24 h) notably inhibited the proliferation of human lung adenocarcinoma cell line A549 via downregulating the key regulatory molecules in the nuclear factor-*κ*B (NF-*κ*B) pathway, including phosphoinositide 3-kinase (PI3K) Class III, protein kinase B (Akt), p-Akt, nuclear factor-*κ*B-inducing kinase (NIK), I*κ*B kinase (IKK) *α*&*β*, nuclear factor *κ*B*α* (I*κ*B*α*), and decreasing the expression of Ki67 (a clinical biomarker for tumor progress evaluation), as well as upregulating the levels of caspase-3. Furthermore, imperialine (10 mg/kg for 18 days) prominently blocked the development of non-small-cell lung cancer in the mice model, which involves the downregulation of the levels of inflammatory cytokines, such as IL-1*β*, IL-6, tumor necrosis factor-*α* (TNF-*α*), and the decrease of Ki67 [50].

#### 5.1.2. Colorectal Cancer

As a major bioactive constituent of FCB, peiminine has been validated to exert promising antitumor effects. Based on the past studies, peiminine (3 mg/kg/2 days for 14 days) could significantly repress the growth of transplantable HCT-116 tumor in mice through inducing apoptosis and autophagy. The molecular mechanism was related to the enhancement of microtubule-associated protein light chain3B (LC3B) and cleaved caspase-3 levels. Analogously, it was discovered that the cell viability was dose-dependently decreased in colorectal carcinoma HCT-116 cells treated with peiminine (0–400 *μ*M for 24 h). The positive results may ascribe the elevation of the ratio of LC3B-II/LC3B-I and the upregulation of phosphorylated Unc51-like kinase 1 (p-ULK1), phosphorylated adenosine 5′-monophosphate-activated protein kinase (p-AMPK), caspase-9, and cleaved caspase-3 levels, as well as the damping of phosphorylated mammalian target of rapamycin (p-mTOR), p-Akt, phosphorylated phosphatase, and tensin homolog (p-PTEN) activities [51]. Furthermore, peiminine (0–400 *μ*M for 48 h) could trigger the death of HCT-116 cells by promoting apoptosis and autophagic flux via modulating the production of metabolites, such as glucose, glutamine, oleate, and lignocerate [52].

#### 5.1.3. Liver Cancer

An accumulating line of evidence indicates that FCB is a potential antitumor agent. In previous studies, various fractions from FCB, mainly including chloroform extracts, n-hexane extracts, water extracts, petroleum ether extracts, and TFCB, have been demonstrated to exhibit inhibitory activity on the growth of the HepG2 [78]. To further evaluate the antitumor activity of FCB, Chao et al. found that peiminine (0–6 *μ*g/mL for 24 h) conducted the growth suppression effects on HepG2 cells in a concentration-dependent manner. It was simultaneously observed that the number of apoptotic cells increased and the percentage of G2/M phases cells augmented. Moreover, the expression of Bax, cleaved poly-ADP-ribosyl-polymerase (PARP), and caspase-3/8/9 increased, whereas the levels of Bcl-2 and Checkpoint kinase 2 (Chk2) downregulated, which indicates that peiminine triggers apoptosis in HepG2 via both extrinsic and intrinsic apoptotic pathways [53].

#### 5.1.4. Endometrial Cancer and Ovarian Cancer

As research continues, some studies have discovered that the FC-AE has huge therapeutic potential in endometrial cancer and ovarian cancer. For instance, Bokhari et al. illustrated that FC-AE (200 *μ*g/mL for 72 h) could markedly repress the growth of endometrial cancer cells through downregulating the transforming growth factor-*β* (TGF-*β*)/Smad signaling pathway [54]. Additionally, another study denoted that FC-AE (200 mg/mL for 96 h) prominently inhibited the viability of ovarian and endometrial cancer cells, which involves the accumulation of S-phase cells and the increase of apoptotic cells. Further investigation showed that FC-AE attenuated the expression of cyclin D1, cyclin D3, NF-*κ*B subunit p50 (NF-*κ*Bp50), p-I*κ*Ba, cysteine-X-cysteine chemokine receptor 4 (CXCR4), and matrix metalloproteinase-9 (MMP-9) and augmented the expression of p27 and caspase-3. The results given above indicate that FC-AE suppresses progression of endometrial cancer and ovarian cancer by activating apoptotic pathways and cell cycle arrest, as well as abrogating the NF-*κ*B pathways activation [55]. Of course, some monomer chemical compositions have also been reported to exhibit advantageous effects on the treatment of endometrial cancer or ovarian cancer. A recent research suggested that peimisine (15 *μ*g/mL for 0–72 h) notably blocked the proliferation of human ovarian cancer cell through inducing apoptosis via increasing G0/G1 phase cell arrest [56].

#### 5.1.5. Other Cancers

Noteworthy, FCB also displays advantageous effects on the treatment of other types of cancers, such as oral cancer and myelogenous leukemia. Based on the published literatures, peimine (50 *μ*g/mL for 3 days) could suppress the proliferation of immortalized and malignant oral keratinocytes through inducing cell cycle arrest and apoptosis. The molecular mechanism was connected with the downregulation of Bcl-2 and retinoblastoma protein (pRb) and the upregulattion of Bax, caspase-3, p53, and p21 protein [57]. Furthermore, Pae et al. has reported the inhibitory effects of peimine on the growth of Human Promyelocytic Leukemia HL-60 Cells [58].

**Figure 8:**
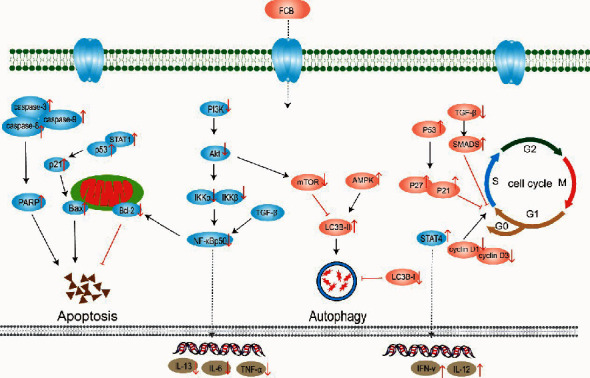
The anticancer mechanism of FCB (“↑” represents the increase; “↓” represents the decrease).

According to the in vitro or in vivo studies mentioned above, FBC or its extracts or its active components exhibit antitumor effects by inducing apoptosis and cell cycle arrest, as well as enhancing autophagic flux. The anticancer mechanism of FCB is shown in Figure 8.

### 5.2. Respiratory Disease

Respiratory diseases are one of the most important causes of morbidity and mortality worldwide [79]. FCB has been wildly used as folk medicine in China for a long time due to its significant therapeutic effects on various respiratory diseases, such as cough, expectoration, pneumonia, bronchial inflammation, and asthma.

#### 5.2.1. Cough

Cough is a crucial defense mechanism which maintains airway patency and eliminates the potential harmful stimuli from the airway and lung [80]. Generally, cough is mostly caused by the common cold [81]. FCB has been used to suppress cough in China for thousands of years. A large number of studies have been carried out to explore the ameliorative effects of FCB on cough. An in vivo study has evaluated the antitussive activities of the alkaloids derived from FCB. Also, the results indicated that imperialine, chuanbeinone, peimine, and peiminine (1.5, 3.0 mg/kg for 1 h) obviously enhanced the latent period of cough and inhibited the cough frequency in mice [59]. Similarly, another research has confirmed that imperialine-*β*-N-oxide, isoverticine, and isoverticine-*β*-N-oxide (1.5–4.5 mg/kg for 1 h) establishe mitigative effects in mice with cough [60]. Additionally, a recent study suggested that the crude alkaloid and FC-AE performed a dose-dependent tracheobronchial relaxation on the carbachol precontracted rat-isolated tracheal and bronchial rings [61]. Moreover, according to the findings of computational target fishing, mitogen-activated protein kinase 1 (MAPK1), Akt1, and PPKCB may be important targets of peimine for the treatment of cough [82].

#### 5.2.2. Expectorant

Expectorant activity is another vital function of FCB. Based on the previous data, the alkaloids extracted from FCB, mainly including imperialine, chuanbeinone, peimine, and peiminine (1.5, 3.0 mg/kg for 0.5 h), have been demonstrated achieve expectorant effects in mice by enhancing tracheal phenol red output [59]. Analogously, Wang et al. illustrated that imperialine-*β*-N-oxide, isoverticine, and isoverticine-*β*-N-oxide (1.5–4.5 mg/kg for 0.5 h) significantly promoted tracheal phenol red output, which indicated that the expectorant effects of FCB may be related to its ability to increase tracheobronchial mucus secretion [60]. The abovementioned results are consistent with the traditional use of FCB as a remedy for expectoration.

#### 5.2.3. Acute Lung Injury

As the research continues, some studies have reported that FCB is a promising agent to treat acute lung injury. TFCB (15–60 mg/kg/day for 5 days) could markedly attenuate LPS-induced acute lung injury in mice, which was associated with a decrease of TNF-*α* and IL-6 [62]. Furthermore, peiminine (0.005 g/kg for 28 days) has been demonstrated to exert inhibitory effects on the bleomycin-induced lung injury rat model by reducing the levels of IFN-*γ*, TGF-*β*, connective tissue growth factor (CTGF), extracellular signal-regulated kinase (ERK)1/2, NF-*κ*B, and Fas Ligand (FasL) [63]. Consistent to the results, another study found that peiminine (0–5 mg/kg for 6 h) attenuated histopathological changes and inhibited the expression of TNF-*α*, IL-1*β*, IL-6, and IL-8 in the LPS-induced acute lung injury mice model. Also, those changes may partly be relevant for the weakening of AKT/PI3K phosphorylation and the attenuation of lipid rafts formation [64].

#### 5.2.4. Chronic Obstructive Pulmonary Disease (COPD)

COPD has long been considered as a severe public health problem. It is urgent to find new compounds for minimizing the risk of this disease [83]. Fortunately, it was shown that imperialine (3.5, 7.0 mg/kg/twice/day for 60 days) could mitigate pulmonary functional and structural impairment in lung tissues of the COPD-like rats. Also, the protective effects were result from the reduction of IL-1*β*, IL-6, IL-8, TNF-*α*, NF-*κ*B p65, TGF-1*β*, MMP-9, and tissue inhibitor of metalloproteinase-1 (TIMP-1) [65]. Besides, peiminine has also been substantiated to prevent the exacerbation of COPD by decreasing the expression of p-AKT and phosphorylated glycogen synthase kinase 3*β* (p-GSK3*β*) while increasing the expression of phosphorylated myosin light chain2 (p-MLC2). Increasingly, an FCB-based traditional Chinese medicine formula, Chuan Bei Pi Pa dropping pills (50–200 mg/kg for 35 days), has been demonstrated to block the progression of COPD through decreasing the number of leukocytes [66].

#### 5.2.5. Asthma

**Figure 9:**
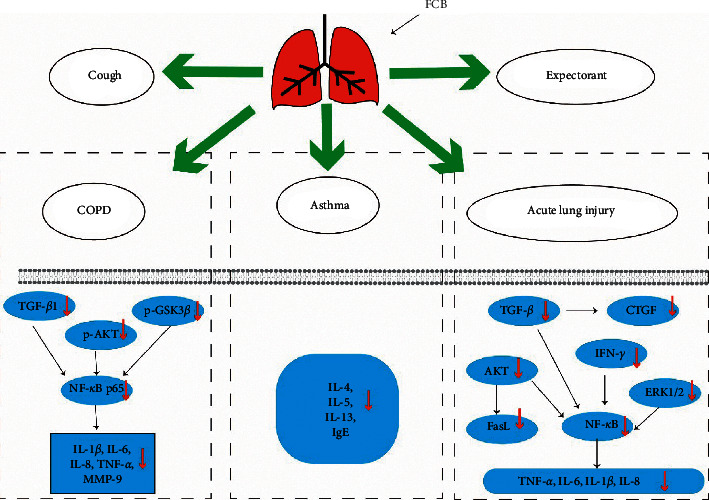
The antirespiratory disease mechanism of FCB (“↓” represents the decrease).

Asthma is a chronic inflammatory disorder of the airways which has been regarded as a formidable challenge for clinicians. At present, the therapeutic effects of conventional medicine are still unsatisfactory [84]. Noteworthily, an in vivo study has indicated that an aqueous extract of FCB (200 mg/kg/three times/week for 56 days) could inhibit the development of asthma in mice by suppressing Th2 cytokines (IL-4, IL-5, and IL-13), IgE, histamine production, and reducing eosinophilic accumulation, as well as increasing of interferon-*γ* production [67] (Figure 9).

### 5.3. Pain

In the previous studies, some research has been conducted to evaluate the analgesic effects of isosteroidal alkaloid from FCB. Peimine as an important ingredient of FCB has been confirmed to exert significant analgesic effects on acetic acid-/formalin-/paclitaxel-induced nociception pain in mice, which indicates that peimine may be a plausible candidate to relieve inflammatory pain and cancer-related neuropathic pain [85].

### 5.4. Anti-Inflammation

**Figure 10:**
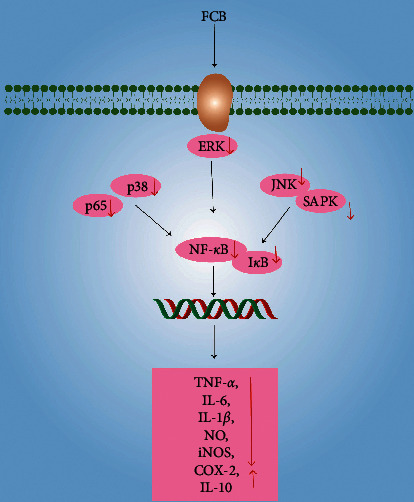
The anti-inflammation mechanism of FCB (“↑”represents the increase, “↓” represents the decrease).

During the past years, the anti-inflammatory effects of FCB have attracted more and more attention. On the grounds of the previous studies, total alkaloid fraction of FCB (0–14 mg/kg/day for 7 days) could significantly attenuate carrageenan-induced paw edema in mice [62]. Consistently, Wang et al. pointed out that the alkaloids isolated from FCB, including imperialine, chuanbeinone, peimine, and peiminine (1.5, 3.0 mg/kg for 0.5 h), could inhibit the xylene-induced mice ear edema in a dose-dependent manner [59]. Moreover, other alkaloids, such as imperialine-*β*-N-oxide, isoverticine, and isoverticine-*β*-N-oxide (1.5–4.5 mg/kg for 0.5 h), also have been found establish similar anti-inflammatory effects [60]. To further investigate the underlying mechanism, Yi et al. suggested that peimine (0–25 mg/L for 18 h) suppressed the inflammatory response through inhibiting the expression of TNF-*α*, IL-6, IL-1*β*, p38, ERK, c-Jun N-terminal kinase (JNK), p65, and I*κ*B and increasing the productions of IL-10 in LPS-induced RAW264.7 macrophages [68]. Additionally, it was demonstrated that peimine or imperialine (0–150 *μ*M for 24 h) could dose-dependently inhibit the expression of nitric oxide (NO), inducible nitric oxide synthase (iNOS), cyclooxygenase-2 (COX-2), TNF-*α*, IL-1*β*, and p–NF–*κ*B p65 in LPS-induced RAW264.7 macrophages [69]. Furthermore, another study has indicated that treatment with the five isosteroid alkaloids derived from FCB (including imperialine, peiminine, delavine, peimisine (0–40 *μ*M for 24 h), and peimine (0–10 *μ*M for 24 h)) could inhibit the productions of NO, TNF-*α*, IL-1*β*, and IL-6 and suppress the activation of ERK1/2, p38 mitogen-activated protein kinase (MAPK), and JNK/stress-activated protein kinase (SAPK) in LPS-induced RAW264.7 macrophages [70] (Figure 10).

### 5.5. Antioxidation

**Figure 11:**
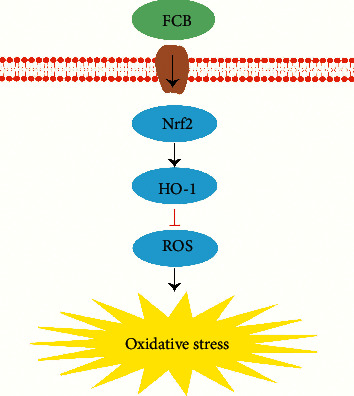
The antioxidation mechanism of FCB (the black arrows represent increase; the red arrows represent decrease).

Based on a recent study, isosteroid alkaloids from FCB (including imperialine, peimine, peiminine, peimisine, imperialine-3-*β*-D-glucoside, and delavine (0–50 *μ*M for 24 h)) could inhibit cigarette smoke-induced oxidative stress in RAW264.7 macrophages, which was associated with a decrease of reactive oxygen species (ROS) and an increase of glutathione, heme oxygenase-1 (HO-1), and nuclear erythroid-related factor 2 (Nrf2), the result indicates that FCB could be a potential inhibitor of oxidative stress [71](Figure 11).

### 5.6. Other Pharmacological Effects

FCB is a natural agent with various pharmacological effects; in addition to the abovementioned pharmacological effects, other biological activities have also been reported in many researches in the last few decades. For instance, peiminine (0–5 mg/kg for 28 days) markedly attenuated behavioural dysfunction and inhibited the loss of dopaminergic neurons and microglial activation in the LPS-induced Parkinson's disease rat model. Moreover, it was ascertained that peiminine (0–50 *μ*g/mLfor 13 h) obviously decreased the expression of the proinflammatory mediators TNF-*α*, IL-6 and IL-1*β*, COX-2, and iNOS by inhibiting the phosphorylation of ERK1/2, AKT, and NF-*κ*B p65 in LPS-induced BV-2 cells [72]. Additionally, peiminine (0–5 mg/kg for 13 h) could exert attenuation effects on LPS-induced mastitis in mice. The positive results were related to downregulation of myeloperoxidase, TNF-*α*, IL-6, IL-1*β*, COX-2, iNOS, AKT, NF-*κ*Bp65, ERK1/2, and p38. Consistent to the results, peiminine (0–70 *μ*g/mL for 4 h) inhibited the secretion of proinflammatory cytokines in LPS-induced mouse mammary epithelial cells via repressing AKT/NF-*κ*B, ERK1/2, and p38 signaling pathways [73]. Based on the result of a recent study, peimine (0–100 *μ*g/mL for 24 h) performed hypoglycemic activities by enhancing the levels of insulin and glucose uptake ability in b-TC6 pancreatic and C2C12 skeletal muscle cells, which implies that peimine has huge therapeutic potential in diabetes [74].

## 6. Pharmacokinetics of FCB

In the past few decades, the pharmacokinetics studies of FCB mainly focused on its alkaloids, such as imperialine, peimisine, and peimine. Imperialine has high oral bioavailability, and a previous study have shown that the bioavailability of administered imperialine at an oral dose of 1 mg/kg, 5 mg/kg, and 10 mg/kg was 31.2%, 53.6%, and 47.4%, respectively [86]. However, the half life of imperialine is short, and if prepared, imperialine is supposed to be taken, at least, at three times a day, which will certainly degrade the patient compliance. Nevertheless, the sustained-release imperialine tablets based on hydroxypropylmethylcellulose could significantly prolong the drug action time and simultaneously improve the oral bioavailability [87]. To further evaluate the intestinal absorption characteristics of imperialine, an in vitro study found that the uptake of imperialine was increased with increasing PH and concentration in Caco-2 cells. Also, some transporters, such as P-glycoprotein and Niemann–Pick C1-Like 1, did not participate in the absorption. Additionally, based on the in situ assessments, the absorption of imperialine varied in four intestinal segments (duodenum, jejunum, ileum, and colon). Among them, the colon displayed the highest level of absorption [88]. Automatically, the pharmacokinetics of peimisine in rats has been assessed by liquid chromatography-tandem mass spectrometry. Also, the results suggested that peimisine was slowly distributed and eliminated from rat plasma and showed linear dynamics in a dose range of 0.26–6.5 mg/kg. Moreover, peimisine was primarily distributed in the spleen, liver, kidney, heart, and lung. Intriguingly, the drug blood and tissue levels in male rats were apparently higher than the female counterparts after oral administration. Moreover, it was confirmed that the major elimination route of male rats was urine excretion after oral peimisine [89]. Similarly, Wu et al. have evaluated the pharmacokinetics, tissue distribution, and excretion of peimine in rats. The results showed that the oral bioavailability in male rats (45.8%) was much higher than in female rats (2.74%). Nevertheless, peimine was metabolized more extensively in female rats than in male rats, and peimine had good tissue penetrability and high tissue affinity in most studied tissues [90]. Furthermore, Chen et al. attempted to investigate the intestinal absorption of peimine in Caco-2 cells. They found that peimine transport was concentration dependent and was increased with increasing PH (6.0–7.4), and P-glycoprotein may participant in the transport of peimine [91]. Some pharmacokinetic parameters of imperialine, peimisine, and peimine are summarized in Table 4, including maximum concentration (*C*_max_), elimination half-life (T1/2), time to reach *C*_max_ (*T*_max_), oral clearance (CL/F), volume distribution/bioavailability (*V*/*F*), and area under the concentration-time curve (AUC) from zero to t and from zero to infinite time.

## 7. Toxicity of FCB

Over the past several millennia, it is generally assumed that FCB is a nontoxic or low toxic herb. To date, few studies focused on the toxicity of FCB. Recently, the acute oral toxicity of FCB has been assessed by an in vivo study. The results revealed that the maximum feasible dose value of FCB in mice was 452.14 g/kg. There were no mice dead after administering the FCB extract at the maximum feasible dose for 14 days. In the histological examination, only the liver showed significant pathological changes [92]. Additionally, Guo et al. have evaluated the toxicity of FCB on human NCM460 colon epithelial cells. It was demonstrated that FC-AE (80 and 160 mg/mL for 72 h) caused mitotic aberrations and genomic instability by inducing spindle assembly checkpoint dysfunction [93]. As the research moves along, it was found that FC-AE (80 and 160 *μ*g/mL for 72 h) leaded to cytokinesis failure through triggering chromosomal instability via promoting the incidence of binucleated cells in NCM460 cells [94]. In summary, the use of FCB should be adopted by appropriate dosage and course of treatment.

## 8. Future Perspectives and Conclusions

FCB is a famous TCM with a wide spectrum of pharmacological effects and has been extensively used as a major ingredient in cough syrups with a huge commercial market. At present, FCB is still mainly harvested from the wild fields. However, the wild *Fritillaria* species used as the botanical origins of FCB grow extremely slow. Also, they are difficult to meet the increasing market demands [95]. In addition, the overexploitation of wild resources results in the extinction of many *Fritillaria* species. Therefore, it is an urgent problem to find out an alternative way to improve the production of FCB. Encouragingly, the emerging of artificial cultivation technique has partly solved the shortage of supplies. Also, the content of total alkaloids in cultivated FCB is higher than that in wild FCB [29]. But, many shortcomings remain; for example, large-scale cultivation of those species is limited because of the restricted growth conditions. Additionally, the output of artificial cultivation is not ideal whereas the cost of that is high, and it still requires a long time to grow into an apparent size [96]. Moreover, the practical value of *Fritillaria* propagation by seed is low because the growth of seedlings in natural habitats is too weak [97]. Recently, tissue culture has been considered as a promising approach because it dramatically reduces the breeding time of *Fritillaria* species. But, there is a fly in the ointment: the stages of rooting and transplanting are difficult, which may result from dormancy of bulbs [98]. Hence, the propagation of *Fritillaria* is another point worth taking into consideration. Of course, searching for the substitutes is also a feasible way to offset the shortage of FCB. For instance, Fritillariae Ussuriensis Bulbus possesses pharmacologic function similar to that of FCB and has abundant natural resources [56,99]. So, it is a valuable subject to verify whether Bulbus Fritillariae Ussuriensis *or* other *Fritillaria* herbs could be used as FCB. Overall, it is necessary to explore the better approach to shorten the growth time of medicinal *Fritillaria* plants, promote the output of FCB, and prevent the overexploitation of wild original species.

In the traditional viewpoint, it is commonly acknowledged that the major bioactive constituents of FCB are alkaloids. Also, a large proportion of pharmacological and chemical studies are focused on them. Moreover, the content of alkaloids has been regarded as an important index to evaluate the qualities of FCB. However, the chemical researches have shown that FCB has the lowest levels of alkaloids compared with other *Fritillaria* species [43]. Therefore, it is a pressing issue to verify whether alkaloids are the most representative of the characteristic pharmaceutical components of FCB. Interestingly, the content of saponins in FCB is higher than any *Fritillaria* species [43], which indicates that saponins may play a pivotal role in the pharmacologic activities of FCB. Hence, it is necessary to conduct more pharmacologic studies to investigate the biological activity of saponins and other compounds from FCB, and more chemical investigations are recommended to concentrate on the other compounds of FCB.

In the past few decades, extensive studies have attempted to assess the pharmacological effects of FCB. To our best knowledge, FCB exerts unexceptionable therapeutic effects on various diseases, including cough, expectoration, acute lung injury, chronic obstructive pulmonary disease, asthma, and some type of cancers, such as lung cancer, colorectal cancer, liver cancer, endometrial cancer, and ovarian cancer. Nevertheless, the underlying mechanisms of its pharmacology actions are still unclear. For instance, the antitussive and expectorant activities have been demonstrated in the previous studies, but the hidden mechanism is not fully understood. Therefore, we should conduct more molecular studies to gain a broader knowledge on the mechanism of FCB's pharmacologic action in future. Additionally, there are not enough studies regarding the pharmacokinetics and toxicity of FCB or its active ingredients. More profound evaluations are required to investigate the pharmacokinetics of other constituents besides alkaloids from FCB. Also, the toxicity studies focusing on molecular levels are still a promising direction.

In the recent years, the growing market demand leads to a rapid rise of the price of FCB. The price of FCB was beyond $260 per kilogram in 2014, almost nine-folds of that in 2004, and it is much higher than other Fritillariae Bulbus [28]. In order to obtain more profits, some merchants adulterate other Fritillariae Bulbus to sell as FCB on commercial markets. However, it is very difficult to distinguish those fakes from FCB by conventional morphological analyses because they have similar morphological characteristics [100, 101]. Recently, some identification methods based on chemical analyses, such as high-performance liquid chromatography (HPLC), have been used to identify and determine isosteroidal alkaloids, which are also inadequate for distinguishing Fritillariae species because the content of chemical compositions is often affected by different practices of planting, harvesting, storage, and manufacturing [102, 103]. Thus, an accurate and efficient identification method should be imminently explored to control the quality of this herb and ensure the drug safety of FCB. Currently, DNA barcodes have been confirmed to be an effective tool for rapid and accurate species discrimination. It could identify plants species by employing a short and standardized DNA region [104, 105]. Based on the previous investigation, ITS2 sequences (a DNA barcode) could successfully distinguish the six original species of FCB from other Fritillariae species. Nevertheless, it is incapable to distinguish the six original species of FCB from each other [106]. Therefore, it is necessary to establish a method for comprehensive differentiation and evaluation of FCB from different varieties in terms of physical properties, chemical compositions, molecular markers, and pharmacological activities.

To sum up, this work is the first to systematically review the traditional uses, sources, phytochemistry, pharmacology, pharmacokinetics, and toxicity of FCB. The recent findings about FCB have been summarized in this work, to provide a reference for further development and utilization of FCB.

## Figures and Tables

**Table 1:** The Chinese patent medicines containing FCB.

Preparation name	Main compositions	Traditional and clinical usages	Reference
Ermu Ningsou Wan	Fritillariae Cirrhosae Bulbus, Anemarrhenae Rhizoma, Gypsum Fibrosum, Gardeniae Fructus, Scutellariae Radix, Mori Cortex, Poria, Trichosanthis Semen, Citri Reticulatae Pericarpium, Aurantii Fructus Immaturus, Glycyrrhizae Radix Et Rhizoma Praeparata Cum Melle, and Schisandrae Sphenantherae Fructus	Clearing and moistening the lung, dissolving phlegm, and relieving cough	[4]
Chuanbei Zhike Lu	Fritillariae Cirrhosae Bulbus, Eriobotryae Folium, Stemonae Radix, Peucedani Radix, Platycodonis Radix, Mori Cortex, and menthol	Dissolving phlegm and relieving cough	[4]
Chuanbei Pipa Tangjiang	Fritillariae Cirrhosae Bulbus, Platycodonis Radix, Eriobotryae Folium, and menthol	Clearing heat, dispersing lung, dissolving phlegm, and relieving cough	[4]
Chuanbei Xueli Gao	Fritillariae Cirrhosae Bulbus, Ophiopogonis Radix, Lilii Bulbus, and Farfarae Flos	Promoting production of body fluid, relieving sore throat, clearing lung, and relieving cough	[4]
Xiao' er Zhisou Tangjiang	Fritillariae Cirrhosae Bulbus, Scrophulariae Radix, Ophiopogonis Radix, Arisaema Cum Bile, Armeniacae Semen Amarum, Arecae Semen Tostum, Platycodonis Radix, Bambusae Caulis In Taenias, Mori Cortex, Trichosanthis Radix, Trichosanthis Semen, Glycyrrhizae Radix Et Rhizoma, Perillae Fructus, Anemarrhenae Rhizoma, and Perillae Folium	Moistening lung, clearing heat, relieving cough, and dissolving phlegm	[4]
Xiao' er Huadu San	Fritillariae Cirrhosae Bulbus, Bovis Calculus Artifactus, Margarita, Realgar, Rhei Radix Et Rhizoma, Coptidis Rhizoma, Glycyrrhizae Radix Et Rhizoma, Trichosanthis Radix, Paeoniae Radix Rubra, Olibanum, Myrrha, and Borneolum Syntheticum	Clearing heat and detoxication, promoting blood circulation, and relieving swelling	[4]
Xiao' er Zhibao Wan	Fritillariae Cirrhosae Bulbus, Perillae Folium, Pogostemonis Herba, Menthae Haplocalycis Herba, Notopterygii Rhizoma Et Radix, Citri Reticulatae Pericarpium, Typhonii Rhizoma, Arisaema Cum Bile, Sinapis Semen, Arecae Semen, Crataegi Fructus, Poria, Medicated Leaven, Hordei Fructus Germinatus, Amber, Borneolum Syntheticum, Gastrodiae Rhizoma, Uncariae Ramulus Cum Uncis, Bombyx Batryticatus, Cicadae Periostracum, Scorpio, Bovis Calculus Artifactus, Realgar, Talcum, and Cinnabaris	Dispelling wind, relieving convulsion, removing stagnation, and dissolving phlegm	[4]
Xiao' er Jindan Pian	Fritillariae Cirrhosae Bulbus, Cinnabaris, Citri Grandis Exocarpium, Arisaema Cum Bile, Peucedani Radix, Scrophulariae Radix, Pinelliae Rhizoma, Isatidis Folium, Akebiae Caulis, Platycodonis Radix, Schizonepetae Spica, Notopterygii Rhizoma Et Radix, Tamaricis Cacumen, Rehmanniae Radix, Aurantii Fructus, Paeoniae Radix Rubra, Uncariae Ramulus Cum Uncis, Puerariae Thomsonii Radix, Arctii Fructus, Gastrodiae Rhizoma, Glycyrrhizae Radix Et Rhizoma, Saposhnikoviae Radix, Borneolum Syntheticum, Bubali Cornu, Saigae Tataricae Cornu, and menthol	Dispelling wind, dissolving phlegm, clearing heat, and detoxication	[4]
Xiao' er Kechuan Keli	Fritillariae Cirrhosae Bulbus, Ephedrae Herba, Armeniacae Semen Amarum, Scutellariae Radix, Bambusae Concretio Silicea, Perillae Fructus, Bombyx Batryticatus, Crataegi Fructus, Raphani Semen, Gypsum Fibrosum, Houttuyniae Herba, Asari Radix Et Rhizoma, Glycyrrhizae Radix Et Rhizoma, Platycodonis Radix, and tea	Clearing heat, dispersing lung, dissolving phlegm, relieving cough, and asthma	[4]
Xiao' er Qingfei Zhike Pian	Fritillariae Cirrhosae Bulbus, Perillae Folium, Chrysanthemi Flos, Puerariae Thomsonii Radix, Armeniacae Semen Amarum, Eriobotryae Folium, Perillae Fructus, Mori Cortex, Peucedani Radix, Belamcandae Rhizoma, Gardeniae Fructus, Scutellariae Radix, Anemarrhenae Rhizoma, Isatidis Radix, Bovis Calculus Artifactus, and Borneolum Syntheticum	Clearing heat, relieving cough, and dissolving phlegm	
Zhisou Huatan Wan	Fritillariae Cirrhosae Bulbus, Papaveris Pericarpium, Platycodonis Radix, Anemarrhenae Rhizoma, Peucedani Radix, Citri Reticulatae Pericarpium, Rhei Radix Et Rhizoma, Glycyrrhizae Radix Et Rhizoma Praeparata Cum Melle, Gypsum Fibrosum, Armeniacae Semen Amarum, Perillae Folium, Descurainiae Semen Lepidii Semen, Farfarae Flos, Stemonae Radix, Scrophulariae Radix, Ophiopogonis Radix, Buddlejae Flos, Asparagi Radix, Schisandrae Chinensis Fructus, Aurantii Fructus, Trichosanthis Semen, Pinelliae Rhizoma, Aucklandiae Radix, Aristolochiae Fructus, and Mori Follum	Clearing lung, dissolving phlegm, relieving cough, and asthma	[4]
Beiling Jiaonang	Fritillariae Cirrhosae Bulbus, Saigae Tataricae Cornu, Hyodeoxycholic acid, Moschus, Aquilariae Lignum Resinatum, Bambusae Concretio Silicea, Chloriti Lapis, and Borax	Clearing heat, dissolving phlegm, relieving cough, and asthma	[4]
Niuhuang Shedan Chuanbei Ye	Fritillariae Cirrhosae Bulbus, Bovis Calculus Artifactus, snake bile, and menthol	Clearing heat, dissolving phlegm, and relieving cough	[4]
Ganlu Xiaodu Wan	Fritillariae Cirrhosae Bulbus, Talcum, Artemisiae Scopariae Herba, Acori Tatarinowii Rhizoma, Akebiae Caulis, Belamcandae Rhizoma, Amomi Fructus Rotundus, Forsythiae Fructus, Scutellariae Radix, Pogostemonis Herba, and Menthae Haplocalycis Herba	Dispelling dampness, clearing heat, and detoxication	[4]
Baihe Gujin Koufuye	Fritillariae Cirrhosae Bulbus, Lilii Bulbus, Rehmanniae Radix, Rehmanniae Radix Praeparata, Ophiopogonis Radix, Scrophulariae Radix, Angelicae Sinensis Radix, Paeoniae Radix Alba, Platycodonis Radix, and Glycyrrhizae Radix Et Rhizoma	Tonifying yin, moistening lung, dissolving phlegm, and relieving cough	[4]
Baihe Gujin Wan	Fritillariae Cirrhosae Bulbus, Lilii Bulbus, Rehmanniae Radix, Rehmanniae Radix Praeparata, Ophiopogonis Radix, Scrophulariae Radix, Angelicae Sinensis Radix, Paeoniae Radix Alba, Platycodonis Radix, and Glycyrrhizae Radix Et Rhizoma	Tonifying yin, moistening lung, dissolving phlegm, and relieving cough	[4]
Baihe Gujin Pian	Fritillariae Cirrhosae Bulbus, Lilii Bulbus, Rehmanniae Radix, Rehmanniae Radix Praeparata, Ophiopogonis Radix, Scrophulariae Radix, Angelicae Sinensis Radix, Paeoniae Radix Alba, Platycodonis Radix, and Glycyrrhizae Radix Et Rhizoma	Tonifying yin, moistening lung, dissolving phlegm, and relieving cough	[4]
Baihe Gujin Keli	Fritillariae Cirrhosae Bulbus, Lilii Bulbus, Rehmanniae Radix, Rehmanniae Radix Praeparata, Ophiopogonis Radix, Scrophulariae Radix, Angelicae Sinensis Radix, Paeoniae Radix Alba, Platycodonis Radix, and Glycyrrhizae Radix Et Rhizoma	Tonifying yin, moistening lung, dissolving phlegm, and relieving cough	[4]
Miaoling Wan	Fritillariae Cirrhosae Bulbus, Notopterygii Rhizoma Et Radix, Scrophulariae Radix, Akebiae Caulis, Menthae Haplocalycis Herba, Paeoniae Radix Rubra, Arisaematis Rhizoma, Rehmanniae Radix, Puerariae Thomsonii Radix, Platycodonis Radix, Pinelliae Rhizoma, Citri Grandis Exocarpium, Uncariae Ramulus Cum Uncis, Peucedani Radix, Borneolum Syntheticum, Cinnabaris, Saigae Tataricae Cornu, and Bubali Cornu	Clearing heat, dissolving phlegm, dispelling wind, and relieving convulsion	[4]
Jinsang Qingyin Wan	Fritillariae Cirrhosae Bulbus, Scrophulariae Radix, Rehmanniae Radix, Ophiopogonis Radix, Scutellariae Radix, Moutan Cortex, Paeoniae Radix Rubra, Alismatis Rhizoma, Coicis Semen, Dendrobii Caulis, Bombyx Batryticatus, Menthae Haplocalycis Herba, Sterculiae Lychnophorae Semen, Cicadae Periostracum, Oroxyli Semen, and Glycyrrhizae Radix Et Rhizoma	Tonifying yin, clearing lung, dissolving phlegm, and relieving sore throat	[4]
Zhike Chuanbei Pipa Diwan	Fritillariae Cirrhosae Bulbus, Eriobotryae Folium, Platycodonis Radix, Pinelliae Rhizoma, and menthol	Clearing heat, dissolving phlegm, and relieving cough	[4]
Zhike Chuanbei Pipa Lu	Fritillariae Cirrhosae Bulbus, Eriobotryae Folium, Platycodonis Radix, Pinelliae Rhizoma, and menthol	Clearing heat, dissolving phlegm, and relieving cough	[4]
Shenrong Baotai Wan	Fritillariae Cirrhosae Bulbus, Codonopsis Radix, Longan Arillus, Cuscutae Semen, Cyperi Rhizoma, Poria, Dioscoreae Rhizoma, Artemisiae Argyi Folium, Atractylodis Macrocephalae Rhizoma, Scutellariae Radix, Rehmanniae Radix Praeparata, Paeoniae Radix Alba, Asini Corii Colla, Glycyrrhizae Radix Et Rhizoma Praeparata Cum Melle, Angelicae Sinensis Radix, Taxilli Herba, Chuanxiong Rhizoma, Notopterygii Rhizoma Et Radix, Dipsaci Radix, Cervi Cornu Pantotrichum, Eucommiae Cortex, Amomi Fructus, and Citri Grandis Exocarpium	Tonifying Qi and Yang and promoting blood circulation to remove blood stasis	[4]
Fufang Chuanbeijing Pian	Fritillariae Cirrhosae Bulbus, Ephedrae Herba, Citri Reticulatae Pericarpium, Platycodonis Radix, Schisandrae Chinensis Fructus, Glycyrrhizae Radix Et Rhizoma, Pinelliae Rhizoma Praeparatum, and Polygalae Radix.	Dispersing lung, dissolving phlegm, relieving cough, and asthma	[4]
Yangyin Qingfei Wan	Fritillariae Cirrhosae Bulbus, Rehmanniae Radix, Ophiopogonis Radix, Scrophulariae Radix, Paeoniae Radix Alba, Moutan Cortex, Menthae Haplocalycis Herba, and Glycyrrhizae Radix Et Rhizoma	Tonifying Yin, moistening and clearing lung, and relieving sore throat	[4]
Yangyinqingfei Koufuye	Fritillariae Cirrhosae Bulbus, Rehmanniae Radix, Ophiopogonis Radix, Scrophulariae Radix, Paeoniae Radix Alba, Moutan Cortex, Menthae Haplocalycis Herba, and Glycyrrhizae Radix Et Rhizoma	Tonifying Yin, moistening and clearing lung, and relieving sore throat	[4]
Yangyin Qingfei Gao	Fritillariae Cirrhosae Bulbus, Rehmanniae Radix, Ophiopogonis Radix, Scrophulariae Radix, Paeoniae Radix Alba, Moutan Cortex, Menthae Haplocalycis Herba, and Glycyrrhizae Radix Et Rhizoma	Tonifying Yin, moistening and clearing lung, and relieving sore throat	[4]
Yangshen Baofei Wan	Fritillariae Cirrhosae Bulbus, Schisandrae Chinensis Fructus, Citri Reticulatae Pericarpium, Amomi Fructus, Aurantii Fructus Immaturus, Ephedrae Herba, Armeniacae Semen Amarum, Gypsum Fibrosum, Glycyrrhizae Radix Et Rhizoma, Panacis Quinquefolii Radix, and Scrophulariae Radix	Tonifying Yin, moistening lung, relieving cough, and asthma	[4]
Yifei Qinghua Gao	Fritillariae Cirrhosae Bulbus, Astragali Radix, Codonopsis Radix, Glehniae Radix, Ophiopogonis Radix, Agrimoniae Herba, Bistortae Rhizoma, Patriniae Herba, Hedyotis Diffusa, Asteris Radix Et Rhizoma, Platycodonis Radix, Armeniacae Semen Amarum, and Glycyrrhizae Radix Et Rhizoma	Tonifying Yin, and Qi, clearing heat and detoxication, resolving phlegm, and relieving cough	[4]
Shedan Chuanbei Ruanjiaonang	Fritillariae Cirrhosae Bulbus and snake bile	Clearing lung, relieving cough, and dissolving phlegm	[4]
Shedan Chuanbei Jiaonang	Fritillariae Cirrhosae Bulbus and snake bile	Clearing lung, relieving cough, and dissolving phlegm	[4]
Shedan Chuanbei San	Fritillariae Cirrhosae Bulbus and snake bile	Clearing lung, relieving cough, and dissolving phlegm	[4]
Qingfei Huatan Wan	Fritillariae Cirrhosae Bulbus, Scutellariae Radix, Armeniacae Semen Amarum, Trichosanthis Semen, Arisaema Cum Bile, Pinelliae Rhizoma Praeparatum, Citri Reticulatae Pericarpium, Poria, Aurantii Fructus, Ephedrae Herba, Platycodonis Radix, Perilla Frutescens, Raphani Semen, Farfarae Flos, and Glycyrrhizae Radix Et Rhizoma	Dissolving phlegm and relieving cough and asthma	[4]
Qingyin Wan	Fritillariae Cirrhosae Bulbus, Chebulae Fructus, Chinese Gall Leaven, Mume Fructus, Puerariae Thomsonii Radix, Poria, Glycyrrhizae Radix Et Rhizoma, and Trichosanthis Radix	Clearing heat, relieving sore throat, producing saliva, and embellish dryness	[4]
Tingbei Jiaonang	Fritillariae Cirrhosae Bulbus, Descurainiae Semen Lepidii Semen, Ephedrae Herba, Armeniacae Semen Amarum, Trichosanthis Pericarpium, Gypsum Fibrosum, Scutellariae Radix, Houttuyniae Herba, Inulae Flos, Haematitum, Ginkgo Semen, Gecko, Platycodonis Radix, and Glycyrrhizae Radix Et Rhizoma	Clearing lung, dissolving phlegm, and relieving cough and asthma	[4]
Biyanling Pian	Fritillariae Cirrhosae Bulbus, Xanthii Fructus, Magnoliae Flos, Angelicae Dahuricae Radix, Asari Radix Et Rhizoma, Scutellariae Radix, Sojae Semen Praeparatum, and menthol	Clearing heat and dispelling wind, detumescence, and relieving rhinitis and rhinobyon	[4]
Juhong Huatan Wan	Fritillariae Cirrhosae Bulbus, Citri Grandis Exocarpium, Physalis Calyx Seu Fructus, Armeniacae Semen Amarum, Papaveris Pericarpium, Schisandrae Chinensis Fructus, Alumen, and Glycyrrhizae Radix Et Rhizoma	Astringing lung, dissolving phlegm, and relieving cough and asthma	[4]
Dianxiankang Jiaonang	Fritillariae Cirrhosae Bulbus, Gastrodiae Rhizoma, Acori Tatarinowii Rhizoma, Bombyx Batryticatus, Arisaema Cum Bile, Salviae Miltiorrhizae Radix Et Rhizoma, Polygalae Radix, Scorpio, Ophiopogonis Radix, Lophatheri Herba, Zingiberis Rhizoma Recens, Amber, Ginseng Radix Et Rhizoma, Borneolum Syntheticum, and Bovis Calculus Artifactus	Relieving convulsion, calming wind, dissolving phlegm, and restoring a clear head	[4]

*Note*. “Dispelling wind” is a therapeutic principle and method of traditional Chinese medicine for cough, rhinitis, and convulsion; “Tonifying yin” and “moistening lung” are the ways to promote the production of body fluid which could prevent the lung from being damaged by harmful substances from outside; Tonifying Qi and Yang is a therapeutic method of traditional Chinese medicine to improve the body's immunity.

**Table 2:** Chemical constituents isolated from FCB.

Name	Molecular formula	Reference
*Alkaloids*		
Imperialine	C_27_H_43_NO_3_	[30]
Peiminine	C_27_H_45_NO_3_	[30]
Peimine	C_27_H_43_NO_3_	[30]
Peimisine	C_27_H_41_NO_3_	[30]
Chuanbeinone	C_27_H_43_NO_2_	[30]
Delavinone	C_27_H_43_NO_2_	[30]
Petilidine	C_27_H_45_NO_2_	[30]
Solanidine 3-O-*α*-L-rhamnopyranosy l-(1 ⟶ 2)-[*β*-D-glucopy ranosyl-(1 ⟶ 4)] -*β*-D-glucopyranoside	C_45_H_73_NO_15_	[30]
Demissidine	C_27_H_45_NO	[30]
Puqienine B	C_28_H_45_NO_3_	[30]
Isodelavine	C_27_H_45_NO_2_	[30]
Isoforticine	C_27_H_45_NO_2_	[30]
Cirrhosinine A	C_27_H_47_NO_4_	[30]
Cirrhosinine B	C_27_H_45_NO_4_	[30]
Demissidine-3-O-*β*-D-glucopyranosyl (1 ⟶ 4)glucopyranoside	C_39_H_65_NO_11_	[30]
Delavidine	C_27_H_39_NO_4_	[30]
Ebeiedinone	C_27_H_43_NO_2_	[30]
Delavine	C_27_H_45_NO_2_	[30]
Ebeiedine	C_27_H_45_NO_2_	[30]
Isoverticine	C_27_H_45_NO_3_	[30]
Sonebeinine	C_27_H_45_NO_2_	[31]
Solanidine	C_27_H_43_NO	[32]
Songbeinone	C_27_H_43_NO_2_	[32]
Songbeisine	C_27_H_41_NO_3_	[32]
Delafrinone	C_27_H_43_NO_3_	[32]
Imperialine-*β*-N-oxide	C_27_H_43_NO_4_	[33]
Ebeinone	C_27_H_41_NO_2_	[33]
Peimisine-3-O-*β*-D-glucopyranoside	C_33_H_51_NO_8_	[34]
Puqiedinone3-O-*β*-D-glucopyranoside	C_33_H_53_NO_7_	[34]
Puqiedinone	C_27_H_43_NO_2_	[34]
Puqiedine	C_27_H_41_NO_2_	[34]
Isoverticine-*β*-N-oxide	C_27_H_45_NO_4_	[35]
(22R,25S)-Solanid-5-enine-3b,5a,6b-triol	C_27_H_45_NO_3_	[11]

*Nucleosides*		
Uracil	C_4_H_4_N_2_O_2_	[30]
Thymine	C_5_H_6_N_2_O_2_	[30]
Cytidine	C_9_H_13_N_3_O_5_	[30]
Inosine	C_10_H_12_N_4_O_5_	[30]
Uridine	C_9_H_12_N_2_O_6_	[30]
Guanosine	C_10_H_13_N_5_O_5_	[30]
Thymidine	C_10_H_14_N_2_O_5_	[30]
Adenosine	C_10_H_13_N_5_O_4_	[30]
Guanine	C_5_H_5_N_5_O	[36]

*Organic acids*		
E-cinnamic acid	C_9_H_8_O_2_	[30]
2-Monopalmitin	C_19_H_38_O_4_	[30]
E-3,4,5-trimethoxycinnamic acid	C_12_H_14_O_5_	[30]
E-*p-*methoxycinnamic acid	C_10_H_10_O_3_	[30]
E-*p*-hydroxycinnamic acid	C_9_H_8_O_3_	[30]
E-p-hydroxycinnamic acid methyl ester	C_10_H_10_O_3_	[30]
Ferulic acid	C_10_H_10_O_4_	[30]
Caffeic acid	C_9_H_8_O_4_	[30]
1-O-feruloylglycerol	C_13_H_16_O_6_	[30]
Glycerin monostearate	C_21_H_42_O_4_	[37]
7-Ketositosterol	C_29_H_48_O_2_	[37]
Stearic acid	C_18_H_36_O_2_	[37]
Palmitic acid	C_16_H_32_O_2_	[37]
Formic acid butyl ester	C_5_H_10_O_2_	[38]
Acetic acid butyl ester	C_6_H_12_O_2_	[38]
Tetradecanoic acid	C_14_H_28_O_2_	[38]
Cis-4-coumaric acid	C_9_H_8_O_3_	[38]

*Sterols*		
*β*-Sitosterol	C_29_H_50_O	[30]
Daucosterol	C_35_H_60_O_6_	[30]
Campesterol	C_28_H_48_O	[38]

*Terpenoids*		
Atractylenolide III	C_15_H_20_O_3_	[30]

**Table 3:** The pharmacological effects of FCB.

Pharmacological effects	Extract/compounds	Study models	Treatment period	Dosage	Mechanisms	Reference
Anticancer (lung cancer)	Total alkaloids	Lewis lung carcinoma cell	0–72 h	30 *μ*g/mL	Inducing apoptosis by promoting the S-phase cell cycle arrest	[47]
Anticancer (lung cancer)	Total alkaloids	Male C57BL/6J mice	10 days	10–40 mg/kg/day	Inhibiting tumor angiogenesis by downregulating CD31; inducing apoptosis through activating caspase-3	[47]
Anticancer (lung cancer)	Aqueous extract	Non-small-cell lung cancer A549 cells	48 h	0–100 *μ*g/mL	Inducing G2/M arrest; inhibiting the expression of Bcl-2 while increasing the expression of Bax, STAT1, and STAT4 protein	[48]
Anticancer (lung cancer)	Aqueous extract	Female SPF mice	20 days	0.2 mL/2.5 mg/mL/2 day	Upregulating the expression of STAT1, STAT4, IFN*γ*, IL-12, caspase-3, and Bax while decreasing Bcl-2 levels	[48]
Anticancer (lung cancer)	Chuanbeinone	Lewis lung carcinoma cells	48 h	5–15 *μ*g/mL	Inducing S-phase arrest; inhibiting the expression of the antiapoptotic Bcl-2 while increasing the expression of proapoptotic protein Bax and caspase-3	[49]
Anticancer (lung cancer)	Chuanbeinone	Male C57BL/6J mice and ICR mice	10 days	10–40 mg/kg/day	Inhibiting tumor angiogenesis via downregulating CD31; inducing apoptosis through activating caspase-3	[49]
Anticancer (lung cancer)	Imperialine	Human lung adenocarcinoma cell line A549	24 h	200 ng/mL	Downregulating the levels of PI3K Class III, Akt, p-Akt, NIK, IKK*α*&*β*, I*κ*B*α*, and Ki67; upregulating the levels of caspase-3	[50]
Anticancer (lung cancer)	Imperialine	Male BALB/c nude mice	18 days	10 mg/kg	Downregulating the levels of IL-1*β*, IL-6, TNF-*α*, and Ki67	[50]
Anticancer (colorectal cancer)	Peiminine	Colorectal carcinoma HCT-116 cells	24 h	0–400 *μ*M	Elevating ratio of LC3B-II/LC3B-I; downregulating the expression of p-mTOR, p-Akt, p-PTEN; upregulating the expression of p-ULK1, p-AMPK, caspase-9, and cleaved caspase-3	[51]
Anticancer (colorectal cancer)	Peiminine	Female BALB/c nude mice	14 days	3 mg/kg/2 days	Upregulating the expression of LC3B and cleaved caspase-3	[51]
Anticancer (colorectal cancer)	Peiminine	Colorectal carcinoma HCT-116 cells	48 h	0–400 *μ*M	Inducing cancer cell apoptosis and autophagy via modulating the production of metabolites (glucose, glutamine, oleate, and lignocerate)	[52]
Anticancer (liver cancer)	Peiminine	HepG2 cells	24 h	0–6 *μ*g/mL	Inducing G2/M phase cells arrest; increasing the expression of Bax, cleaved PARP, and caspase-3,8,9; decreasing the expression of Bcl-2 and Chk2	[53]
Anticancer (endometrial cancer)	Aqueous extract	Human endometrial cancer cell lines Ishikawa and HEC-1B	72 h	200 *μ*g/mL	Decreasing the expression of TGF-*β*, TGF-*β*R1, TGF-*β*R2, SMADS, *α*v*β*3, MMP-2, MMP-9, FAK, snail, and slug	[54]
Anticancer (endometrial cancer and ovarian cancer)	Aqueous extract	Human ovarian surface epithelial, HOSE 642, and ovarian cancer cell lines OVCA 420 and OVCA 429	96 h	200 mg/mL	Inducing S-phase cell cycle arrest by decreasing expression of cyclin D1, D3; increasing expression of p27 Inhibiting the expression of NF-*κ*Bp50, p-I*κ*Ba, CXCR4 and MMP-9; increasing the expression of caspase-3	[55]
Anticancer (ovarian cancer)	Peimisine	Human ovarian cancer cell line (A2780)	0–72 h	15 *μ*g/mL	Inducing apoptosis by increasing G0/G1 phase cell arrest	[56]
Anti-cancer (oral cancer)	Peimine	Normal human oral keratinocytes (NHOK) and gingival fibroblasts (GF)	3 days	50 *μ*g/mL	Downregulated the expression of Bcl-2 and pRb; upregulated the expression of Bax, caspase-3, p53, and p21	[57]
Anticancer (myelogenous leukemia)	Peimine	Human Promyelocytic Leukemia HL-60 Cells	4 days	2.5, 5.0 mM	—	[58]
Antirespiratory disease (cough)	Imperialine, chuanbeinone, peimine, and peiminine	Kunming mice	1 h	1.5, 3.0 mg/kg	Enhancing the latent period of cough and inhibiting the cough frequency	[59]
Antirespiratory disease (cough)	Imperialine, imperialine-*β*-N-oxide, isoverticine, and isoverticine-*β*-N-oxide	Kunming mice	1 h	1.5, 3.0, 4.5 mg/kg	Enhancing the latent period of cough and inhibiting the cough frequency	[60]
Antirespiratory disease (cough)	Crude alkaloid and water extracts	The rat bronchi and tracheas	10-9-10–5 g/mL 10-7-10–3 g/mL	5–7 min	—	[61]
Antirespiratory disease (expectorant)	Imperialine, chuanbeinone, peimine, and peiminine	Kunming mice	0.5 h	1.5, 3.0 mg/kg	Enhancing tracheal phenol red output	[59]
Antirespiratory disease (expectorant)	Imperialine, imperialine-*β*-N-oxide, isoverticine, and isoverticine-*β*-N-oxide	Kunming mice	0.5 h	1.5, 3.0, 4.5 mg/kg	Enhancing tracheal phenol red output	[60]
Antirespiratory disease (acute lung injury)	Total alkaloid	C57BL/6J mice	5 days	15–60 mg/kg/day	Decreasing the levels of TNF-*α* and IL-6	[62]
Antirespiratory disease (acute lung injury)	Peiminine	Sprague Dawley (SD) rats	28 days	0.005 g/kg	Decreasing the levels of IFN-*γ* in serum and TGF-*β*, CTGF, ERK1/2, NF-*κ*B, and FasL in lung tissue	[63]
Antirespiratory disease (acute lung injury)	Peiminine	Male BALB/c mice	6 h	0–5 mg/kg	Inhibiting the expression of TNF-*α*, IL-1*β*, IL-6, IL-8, and AKT/PI3K; attenuating lipid rafts formation	[64]
Antirespiratory disease (chronic obstructive pulmonary disease)	Imperialine	Wistar rats	60 days	3.5, 7.0 mg/kg/twice/day	Decreasing the levels of IL-1*β*, IL-6, IL-8, TNF-*α*, NF-*κ*B p65, TGF-*β*1, MMP-9, and TIMP-1	[65]
Antirespiratory disease (chronic obstructive pulmonary disease)	Chuan Bei Pi Pa dropping pills	Male Kunming mice	35 days	50–200 mg/kg	Decreasing the number of leukocytes	[66]
Antirespiratory disease (chronic obstructive pulmonary disease)	Peiminine	HBSMC cells	0-1 h	10 *μ*M	Decreasing the expression of p-AKT and p-GSK3*β*; increasing the expression of p-MLC2	[66]
Antirespiratory disease (asthma)	Aqueous extract	Male C57BL/6 mice	56 days	200 mg/kg/three times/week	Suppressing Th2 cytokines (IL-4, IL-5, and IL-13), IgE, and histamine production; reducing eosinophilic accumulation; and increasing of interferon-*γ* production	[67]
Anti-inflammation	Total alkaloids	SD rats	7 days	0–14 mg/kg/day	—	[62]
Anti-inflammation	Imperialine, chuanbeinone, peimine, and peiminine	Kunming mice	0.5 h	1.5, 3.0 mg/kg	—	[59]
Anti-inflammation	Imperialine, imperialine-*β*-N-oxide, isoverticine, and isoverticine-*β*-N-oxide	Kunming mice	0.5 h	1.5, 3.0, 4.5 mg/kg	—	[60]
Anti-inflammation	Peimine	RAW264.7 macrophages	18 h	0–25 mg/L	Inhibiting the expression of TNF-*α*, IL-6, IL-1*β*, p38, ERK, JNK, p65, and IkB; increasing the expression of IL-10	[68]
Anti-inflammation	Imperialine and peimine	RAW264.7 macrophages	24 h	0–150 *μ*M	Decreasing the expression of NO, iNOS, COX-2, TNF-*α*, IL-1*β*, and P-NF-*κ*B p65	[69]
Anti-inflammation	Imperialine, peiminine, delavine, peimisine, and peimine	RAW264.7 macrophages	24 h	0–40 *μ*M 0–10 *μ*M	Decreasing the expression of NO, TNF-*α*, IL-1*β*, IL-6, ERK1/2, p38 MAPK, and JNK/SAPK	[70]
Antioxidation	Imperialine, peimine, peiminine, peimisine, imperialine-3-*β*-D-glucoside, and delavine	RAW264.7 macrophages	24 h	0–50 *μ*M	Decreasing the levels of ROS; increasing the levels of glutathione, HO-1, and Nrf2	[71]
Parkinson's disease	Peiminine	Female Wistar rats	28 days	0–5 mg/kg	—	[72]
Parkinson's disease	Peiminine	BV-2 cells	13 h	0–50 *μ*g/mL	Decreasing the expression of TNF-*α*, IL-6, IL-1*β*, COX-2, iNOS, ERK1/2, AKT, and NF-*κ*B p65	[72]
Mastitis	Peiminine	BALB/c mice	13 h	0–5 mg/kg	Inhibiting the expression of myeloperoxidase, TNF-*α*, IL-6, IL-1*β*, COX-2, iNOS, AKT, NF-*κ*Bp65, ERK1/2, and p38	[73]
Mastitis	Peiminine	Mouse mammary epithelial cells (mMECs)	4 h	0–70 *μ*g/mL	Inhibiting the expression of TNF-*α*, IL-6, IL-1*β*, COX-2, iNOS, AKT, NF-*κ*Bp65, ERK1/2, and p38	[73]
Diabetes	Peimine	b-TC6 pancreatic and C2C12 skeletal muscle cells	24 h	0–100 *μ*g/mL	Increasing the levels of insulin secreted into the media and glucose uptake ability	[74]

**Table 4:** Pharmacokinetics of FCB.

Study models	Compounds	Dosage	Method of administration	*T*_1/2_ (h)	*T*_max_ (h)	CL/F (L·kg/h)	V\F (L/kg)	*C*_max_ (*μ*g/L)	AUC_0–t_*μ*g h/L)	AUC_0–∞_*μ*g h/L)	Reference
Male Sprague Dawley (SD) rats	Imperialine	5 mg/kg	i.v.	14.731 ± 19.393	—	2.087 ± 0.666	—	—	1105.206 ± 1019.526	2590.930 ± 820.813	[86]
Male SD rats	Imperialine	1 mg/kg	i.g.	1.906 ± 0.703	0.467 ± 0.347	6.310 ± 0.971	—	60.929 ± 17.356	156.466 ± 26.218	161.672 ± 26.129	[86]
Male SD rats	Imperialine	5 mg/kg	i.g.	4.683 ± 4.409	0.550 ± 0.274	3.955 ± 1.151	—	318.019 ± 62.449	1179.344 ± 238.451	1388.993 ± 546.863	[86]
Male SD rats	Imperialine	10 mg/kg	i.g.	2.305 ± 0.961	0.600 ± 0.224	4.228 ± 0.924	—	743.601 ± 234.293	2287.713 ± 406.720	2455.225 ± 522.966	[86]
Beagle dogs	Imperialine	5 mg	i.g.	3.041 ± 1.928	1.167 ± 0.258	—	—	12.335 ± 11.629	36.430 ± 5.944	40.213 ± 10.554	[87]
Beagle dogs	Imperialine tablet	5 mg	i.g.	11.890 ± 13.622	4.333 ± 1.366	—	—	7.723 ± 1.166	89.581 ± 21.243	91.170 ± 20.997	[87]
SD rats	Peimine	1 mg/kg	i.g.	♀2.86 ± 1.29	♀0.67 ± 0.31	♀67.0 ± 11.6	♀280 ± 160	♀3.67 ± 1.13	♀14.5 ± 3.27	♀15.3 ± 3.06	[90]
				♂2.78 ± 0.85	♂0.70 ± 0.11	♂6.53 ± 2.35	♂25.5 ± 9.07	♂38.9 ± 9.34	♂159 ± 50.1	♂167 ± 52.5	

SD rats	Peimine	2 mg/kg	i.g.	♀4.05 ± 2.48	♀0.40 ± 0.36	♀113 ± 33.5	♀649 ± 419	♀7.45 ± 7.10	♀17.3 ± 5.40	♀18.8 ± 5.01	[90]
				♂3.05 ± 0.78	♂1.45 ± 1.42	♂4.40 ± 0.98	♂19.4 ± 6.70	♂94.8 ± 17.1	♂446 ± 111	♂475 ± 119	

SD rats	Peimine	4 mg/kg	i.g.	♀1.84 ± 0.58	♀0.40 ± 0.35	♀152 ± 28.2	♀387 ± 74.2	♀12.6 ± 5.14	♀26.6 ± 5.84	♀27.1 ± 5.78	[90]
				♂2.33 ± 0.86	♂0.70 ± 0.11	♂5.59 ± 1.48	♂18.6 ± 9.14	♂176 ± 45.2	♂737 ± 236	♂769 ± 257	

SD rats	Peimine	0.4 mg/kg	i.v.	—	—	♀4.47 ± 1.48	♀82.5 ± 70.3	—	♀65.6 ± 7.03	♀98.9 ± 35.8	[90]
						♂2.43 ± 0.37	♂10.9 ± 1.56		♂159.5 ± 20.2	♂167.6 ± 23.0	

SD rats	Peimisine	0.26 mg/kg	i.g.	♀3.2 ± 1.8	♀4.0 ± 0.0	♀9.6 ± 3.9	♀43.8 ± 27.2	♀5.6 ± 2.3	♀26.1 ± 9.7	♀30.0 ± 10.9	[89]
				♂3.9 ± 1.7	♂4.0 ± 0.0	♂2.2 ± 0.02	♂12.5 ± 5.7	♂17.8 ± 1.7	♂102.3 ± 6.5	♂117.2 ± 1.2	

SD rats	Peimisine	1.3 mg/kg	i.g.	♀3.1 ± 1.3	♀3.0 ± 1.4	♀2.8 ± 0.99	♀7.9 ± 2.4	♀58.2 ± 14.7	♀478.3 ± 162.0	♀480.0 ± 164.4	[89]
				♂3.0 ± 0.26	♂4.0 ± 0.0	♂1.34 ± 0.07	♂5.8 ± 0.22	♂123.6 ± 36.4	♂964.4 ± 46.5	♂970.0 ± 49.1	

SD rats	Peimisine	6.5 mg/kg	i.g.	♀4.8 ± 1.1	♀4.0 ± 0.0	♀5.1 ± 1.7	♀33.8 ± 7.5	♀181.2 ± 32.4	♀1305.7 ± 404.9	♀1389.6 ± 476.2	[89]
				♂4.5 ± 1.3	♂3.5 ± 1.0	♂1.2 ± 0.40	♂7.7 ± 2.4	♂601.7 ± 212.0	♂5529.5 ± 1628.2	♂5695.8 ± 1372.0	

## Abbreviations

AKT: Protein kinase B
Bax: B-cell lymphoma-2-associated X apoptosis regulator
Bcl-2: B-cell lymphoma-2
CD31: Endothelial cell adhesion molecule-1
Chk2: Checkpoint kinase 2
COX-2: Cyclooxygenase-2
CPM: Chinese patent medicines
CTGF: Connective tissue growth factor
CXCR4: Cysteine-X-cysteine chemokine receptor 4
ERK: Extracellular signal-regulated kinase
FasL: Fas Ligand
FC: *Fritillaria cirrhosa* D. Don
FCB: *Fritillariae cirrhosae* Bulbus
FC-AE: Aqueous extract of FCB
FD: *Fritillaria delavayi* Franch
FP: *Fritillaria przewalskii* Maxim
FT: *Fritillaria taipaiensis* P. Y. Li
FTB: *Fritillariae thunbergii* Bulbus
FU: *Fritillaria unibracteata* Hsiao et K. C. Hsia
FW: *Fritillaria unibracteata* Hsiao et K. C. Hsia var. *wabuensis* (S. Y. Tang et S. C. Yue) Z. D. Liu., S. Wang et S. C. Chen; HO-1, heme oxygenase-1
IFN-*γ*: Interferon gamma
IL: Interleukin
IKK: I*κ*B kinase
I*κ*B*α*: Nuclear factor *κ*B*α*
iNOS: Inducible nitric oxide synthase
JNK: c-Jun N-terminal kinase
LC3B: Microtubule-associated protein light chain3 beta
LLC: Lewis lung carcinoma
MAPK: Mitogen-activated protein kinase
MMP-9: Matrix metalloproteinase-9
NF-*κ*Bp50: NF-*κ*B subunit p50
NF-*κ*B: Nuclear factor-*κ*B; NIK, nuclear factor-*κ*B-inducing kinase
NO: Nitric oxide
Nrf2: Nuclear erythroid-related factor 2
PARP: Poly-ADP-ribosyl-polymerase
PI3K: Phosphoinositide 3-kinase
p-AMPK: Phosphorylated adenosine 5′-monophosphate-activated protein kinase
p-ULK1: Phosphorylated Unc51-like kinase 1
p-mTOR: Phosphorylated mammalian target of rapamycin
p-PTEN: Phosphorylated phosphatase and tensin homolog
pRb: Retinoblastoma protein
p-GSK3*β*: Phosphorylated glycogen synthase kinase 3*β*
p-MLC2: Phosphorylated myosin light chain2
ROS: Reactive oxygen species
SAPK: Stress-activated protein kinase
STAT: Signal transducer and activator of transcription
TFCB: Total alkaloids of FCB
TNF-*α*: Tumor necrosis factor-*α*
TIMP-1: Tissue inhibitor of metalloproteinase-1
TGF-*β*: Transforming growth factor-*β*
TCM: Traditional Chinese medicine.
